# Prospective Study to compare Intra-articular versus Intravenous Tranexemic Acid in reducing Post-operative Blood Loss in staged bilateral Total Knee Arthroplasty

**DOI:** 10.5704/MOJ.1611.020

**Published:** 2016-11

**Authors:** N Balasubramanian, GB Natarajan, s Prakasam

**Affiliations:** Department of Orthopaedic Surgery, Saveetha Medical College & University, Chennai, India

**Keywords:** Total knee arthroplasty, Intravenous, Intra-articular, tranexamic acid

## Abstract

The number of total knee arthroplasties (TKA) performed is around two million annually worldwide and this number is expected to increase fivefold by 2025. The most common indication is osteoarthritis of the knee. Blood loss is significant during the post-operative period and blood transfusion when necessary has its own drawbacks. The use of intravenous tranexamic acid has significantly reduced blood loss. We analysed 35 patients who underwent staged bilateral TKA between August 2013 and February 2016 and had administered intra-articular tranexamic acid for one knee and intravenous tranexamic acid for the other knee. The results were analysed based on post-operative blood loss, change in haemoglobin (Hb) level and haematocrit (PCV) and the need for blood transfusion. The average postoperative blood loss was 129.57 ml and 277.71 ml for intra articular group and intravenous group respectively. A control group (no drug or placebo group) with age matched patients (n= 21) was chosen from medical records. The average blood loss in the control group was 493.81 ml. The fall in Hb level and PCV was 0.72 gm/dl and 2.62 % (Intra-articular Group), 1.36 gm/dl and 4.34 % (Intravenous Group) and 2.62 gm/dl and 5.52 % (Control). The number of transfusions were two (Intra-articular Group), five (Intravenous Group) and nine (Control). We conclude that when compared with intravenous route, intra-articular administration has significantly reduced blood loss, Hb level and PCV fall and the rate of blood transfusion.

## Introduction

Total knee arthroplasty (TKA) is the most commonly performed joint replacement surgery worldwide. With an estimated two million surgeries every year worldwide this number is set to increase fivefold by 2025. The major beneficial effects are pain relief, increase range of movements and better quality of life. The most common indication is tri-compartmental arthritis of the knee either primary (idiopathic) or secondary (rheumatoid, post-traumatic, post-septic, etc). The possible complications include post-operative blood loss necessitating transfusion, infection and implant loosening. The cause of potential blood loss in the post-operative period is the extensive bone resection and soft tissue release during surgery. This results in bleeding raw areas which can continue bleeding during the post-operative period. This causes a drop in the blood haemoglobin level and haematocrit concentration which, if significant, requires blood transfusion with potential transfusion reaction^1-3^.

Tranexemic acid (an anti-fibrinolytic agent) has been used to control bleeding during and after pregnancy and in other bleeding disorders^5^. In the past, surgeons have used intravenous tranexamic acid during TKA and have found a significant reduction in blood loss and transfusion numbers post-operatively. The intravenous route can have thrombotic events as systemic effects as reported by a few authors^4^. The local application of tranexamic acid into the knee joint could potentially have the same beneficial effects but without the systemic complications of thrombotic events^6,7,13,14^. However data supporting the intra-articular use of tranexamic acid is limited. We therefore conducted a study to compare intraarticular versus intravenous route of tranexamic acid administration in staged bilateral TKA’s to determine if there is significant difference between the two modes of administration.

## Materials and Methods

**Fig. 1:**
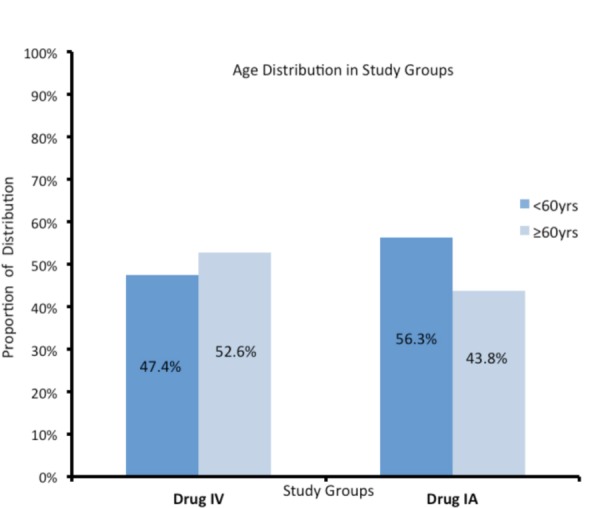
Showing age distribution.

**Fig. 2:**
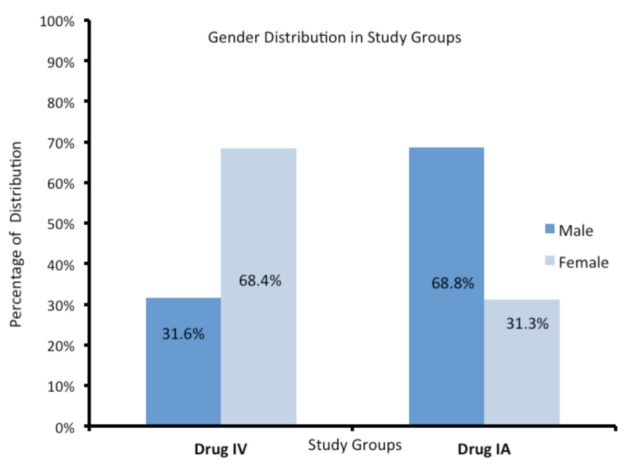
Showing showing gender distribution.

Thirty-five patients with bilateral osteoarthritis knee who underwent staged bilateral TKA were included in this study. There were 17 men and 18 women with average age of 60.17 years (range: 54-68 years). The patient demographic data is shown in Fig. 1 and Fig. 2. We routinely perform TKA for stages 3 and 4 osteoarthritis, with blood pressure ≤140/90 mm Hg. Oral anti-coagulants were stopped one week prior to surgery and for elective procedures the cut-off value of haemoglobin was ≥10 gm/dl as per our standardised department selection criteria.

Both knees were operated within one year of each other (range: one month-11 months). Informed consent was obtained in all our patients and strict ethical guidelines were followed. The patients were randomly allocated into two groups: one receiving intra-articular Tranexamic acid [Trapic 2gm in 20 ml] (Sun Pharmaceuticals™ India) and intravenous 20 ml saline and the other receiving Trapic [2 gm in 20 ml] intravenously and 20 ml saline intra-articular injection for the first knee. The injection of 20 ml saline was as a placebo. Both drug and saline were given at the same time just after closure of the wound through the drain and intravenously. For the second knee surgery, the route of administration was reversed (those who received intraarticular drug for the first knee were given intravenous drug for the second knee and vice versa). A control group of age matched patients (n=21) were chosen from our hospital records who had not been administered tranexamic acid or saline.

All surgeries were done under tourniquet control and the tourniquet was released after wound closure and sterile compression dressing. Suction drain was activated three hours after surgery by the ward staff. The minimum time between the two surgeries was one month and all patients underwent the second knee replacement surgery within one year of the first knee to avoid any new complicating biological comorbidities. All TKAs were done by a single surgical team under combined spinal and epidural anaesthesia using the same implant, namely PFC Sigma [Depuy-Synthes, USA]. The post-operative results were recorded and tabulated with respect to post-operative blood loss, reduction in Hb % level and PCV from pre-op status and the need for blood transfusion. Post op Hb of ≤ 9.0 gm% was the threshold for blood transfusion according to standard surgical protocol of the department. All patients were mobilised full weight bearing on the second post-operative day as well as the restarting of oral anti-coagulants.

## Result

The average duration of surgery in all three groups was comparable, 83.8 ± 8.4 minutes. Sixteen out of 35 knees had intra-articular tranexamic acid for the first knee surgery and 19 were administered intravenous tranexamic acid for the opposite knee. Correspondingly 19 patients had saline injection intravenously for the first knee. The observations were recorded and tabulated under pre and post-op Hb, PCV and fall in their values and volume of blood collected through the suction drain over the first 48 hours postoperatively. Blood transfusions both during and after surgery were also duly noted (Table I).

**Table I:** Showing mean of variables

Mean factors	Control (n=21)	Intra-articular group (n=35)	Intravenous group (n=35)
Average age (years)	59.67	60.17	60.17
Duration of surgery (minutes)	81.3 ± 9.6	84.8 ± 9.1	86.3 ± 8.5
Blood loss during surgery (ml)	140 ± 15	155 ± 10	150 ± 20
Drain over 48 hours (ml)	493.81 ± 134	129.57 ± 98	277.71 ± 74
Fall in Hb (gm/dl)	2.64 ± 1.32	0.72 ± 0.64	1.36 ± 0.72
Fall in PCV (%)	5.52 ± 2.92	2.62 ± 2.52	4.34 ± 2.88
No. of transfusions	9 (25.7%)	2 (0.05%)	5 (14.2%)
(Units of Packed cells)			

The average blood loss during surgery in all three groups was comparable (average of 155 ± 15 ml).However during the post-operative period it was noted that the average volume of blood over 48 hours in the collection container was 129.57 ± 98 ml, 277.71 ± 74 ml and 493.81 ± 134 ml (mean ± 2 S.D) for the intra-articular, intravenous, and control group respectively. Consequently, the fall in post-op Hb level and associated PCV was 0.72 ± 0.64 gm/dl and 2.62 ± 2.5 % (intra-articular) , 1.36 ± 0.72 gm/dl and 4.34 ± 2.8 % (intravenous) and 2.62 ± 1.28 gm/dl and 5.52 ± 2.92 % (control group) . The observations were then subjected to Chi-square analysis and the results were found to be statistically significant with p value < 0.00001 (Table II and III).

**Table II:** Showing drop in Hb levels

	Control	Intra-articular group	Intravenous group	
Hb drop ≤ 1gm/dl	0	26	5	
	(7.15) [7.15]	(11.92) [11.62]	(11.92) [4.02]	
Hb drop > 1 gm/dl	21	9	30	
	(13.85) [3.70]	(23.08) [8.59]	(23.08) [2.08]	
			Chi-square statistic = 42.1535	P value < 0.00001 (Significant)

Chi-square table showing drop in haemoglobin between three groups. The resulting ’P’ value at < 0.00001 is very significant

**Table III:** Showing fall in PCV values

	Control	Group A	Group B	
PCV fall ≤ 3 %	3	27	9	
	(9.00) [4.00]	(15.00) [9.60]	(15.00) [2.40]	
PCV fall > 3 %	18	8	26	
	(12.00) [3.00]	(20.00) [7.20]	(20.00) [1.80]	
			Chi-square statistic = 28	P value < 0.00001 (Significant)

Chi-square table showing drop in haematocrit values at 48 hours . The resulting ’p’ value at < 0.00001 is very significant

Blood transfusion in our centre was started if post-operative Hb level at 48 hours ≤ 9 gm%. There were two transfusions in the intra-articular group (0.05 %), five in intravenous group (14.2%) and nine in the control group (25.7%). There were no thrombotic events in any of our patients.

## Discussions

TKA has become a common surgical practice in the present time. The number is on the rise due to greater patient awareness, better implant designs and increased frequency for performing TKA. However it is associated with increased intraoperative and post-operative bleeding and this requires blood transfusions^1,2,3^. Tranexamic acid by its anti-fibrinolytic action stabilises blood clots and reduces bleeding. Intravenous Tranexamic acid has been shown to significantly reduce bleeding in TKA^4,15^.

Local infiltration of tranexamic acid has been shown to reduce bleeding in patients with bleeding diathesis in dental procedures^5^. Intra-articular tranexamic acid in TKA has been shown to reduce bleeding in the post-operative period with higher haemoglobin and haematocrit value^6,7,13,14^. Craik *et al*^8^ conducted a randomised trial using intra-articular tranexamic acid but they did not use suction drains in any of their patients. They reported reduced bleeding through analysing post-operative Hb and PCV values and concluded that topical tranexamic acid significantly reduces bleeding.

Fernando *et al*^16^ conducted a random controlled trial (RCT) on 50 patients to assess the efficacy of intravenous tranexemic acid in TKA. They reported a 42 % reduction in drain volume (326ml in study vs 626 ml in control) (p : <.001). They also reported that the Hb and PCV levels were significantly higher in patients in whom tranexamic acid was given. There were eight transfusions in control versus none in study group. However they did not use tourniquet in any of their patients.

Digas *et al*^9^ conducted a RCT with 90 patients in three groups (placebo, intra-articular and intravenous tranexamic acid) and reported that the mean drained blood loss in control, IV and intra-articular groups was 415 ± 24, 192 ± 21 and 121 ± 17 ml, respectively which is comparable with our results although in our study the group which were administered intravenous drug had slightly higher bleeding at 277.71 ± 74 ml. Also in our study we reported a lower transfusion rate in both groups (0.05% and 14%) as against (17% and 23%). This could probably be due to different thresholds for blood transfusions in different centres.

Recently published meta-analyses have also shown significant reduction in post-operative blood loss following tranexamic acid when compared to placebo. However one meta-analysis^11^ shows significant difference between topical and intravenous routes but the other authors^12^ reported no significant difference between the two groups.

When compared with literature our study assumes significance in that it is the only study wherein the comparison between intra articular and intravenous was conducted and the results are promising since the same patient was administered tranexamic acid at different times through two different drug routes. This effectively eliminated any confounding variable which could influence the result of the study. The results of our study have clearly shown that tranexamic acid has a definitive role in TKA and between the two routes, intra articular route has significant benefits in reducing post-operative bleeding and blood transfusion rates.

## Conclusion

The number of TKA procedures will grow exponentially over the next few decades. The short term risks of bleeding and subsequent transfusions will also show a similar trend. The role of tranexamic acid has been proven beyond doubt in reducing these complications. Topical use of tranexamic acid may soon replace intravenous route as the established mode of delivery to achieve lower bleeding and transfusion rates.
